# Phytochemical Study and Anti-inflammatory, Antidiabetic and Free Radical Scavenger Evaluations of *Krameria pauciflora* Methanol Extract ^†^

**DOI:** 10.3390/molecules17010861

**Published:** 2012-01-17

**Authors:** M. Ángeles Ramírez-Cisneros, María Yolanda Rios, Myrna Déciga-Campos, A. Berenice Aguilar-Guadarrama

**Affiliations:** 1 Centro de Investigaciones Químicas, Universidad Autónoma del Estado de Morelos, Avenida Universidad No. 1001 Col. Chamilpa, 62209 Cuernavaca, Morelos, Mexico; Email: angelesrc@uaem.mx (M.Á.R.-C.); myolanda@uaem.mx (M.Y.R.); 2 Sección de Estudios de Posgrado e Investigación, Escuela Superior de Medicina, Instituto Politécnico Nacional, Plan de San Luis y Díaz Mirón, México D.F., 11340, Mexico; Email: mdeciga@ipn.mx

**Keywords:** *Krameria pauciflora*, anti-inflammatory, antidiabetic, cycloartanes, catechins

## Abstract

The plant *Krameria pauciflora* MOC et. Sessé ex DC. is used as an anti-inflammatory and antidiabetic in traditional medicine. The aim of this study was to evaluate the *in vivo* anti-inflammatory and antidiabetic effects of a methanol extract from the roots of *K. pauciflora*. Dichloromethane and ethyl acetate extracts obtained by partitioning the methanol extract were also evaluated. Complete methanol and dichloromethane extracts showed anti-inflammatory effects at 3 mg/kg. An anti-inflammatory effect similar to indomethacin (10 mg/kg) was observed when the methanol and dichloromethane extracts, which contain a cycloartane-type triterpene and an sterol, were administered orally at several doses (3, 10, 30 and 100 mg/kg), whereas no anti-inflammatory effect was observed at any dose for the ethyl acetate extract, which contains catechin-type flavonoids. The antidiabetic effect of each extract was also determined. An antihyperglycaemic effect was observed in diabetic rats, but no effect in normoglycaemic animals was observed when the methanol extract was administrated at 30 mg/kg. All of the extracts exhibited radical scavenger activity. Additionally, constituents from all of the extracts were identified by NMR. This article supports the use of *K. pauciflora* as an anti-inflammatory because it exhibits a similar effect to indomethacin. However, its antidiabetic effect is not completely clear, although it could be useful for preventing diabetic complications.

## 1. Introduction

Non-steroidal anti-inflammatory drugs are one of the best-selling groups of drugs globally. Inflammation is a part of the immune response against infection and has been implicated in a broad range of diseases, including diabetes, cancer, hypertension and atherosclerosis [1,2,3,4]. There are 346 million people around the World suffering from diabetes [5]. Throughout history, people have used medicinal plants to treat diseases and illness such as diabetes. For example, there are more than 13,500 reports on the anti-inflammatory effects of plant extracts and more than 4,200 articles about the antidiabetic effects of vegetal extracts. It is interesting to note that some antidiabetic species also exhibited an anti-inflammatory effect and *vice versa* [6,7]. Rhatany is the name given to several species of *Krameria*, a genus distributed from the southeast of the United States to the northern parts of Argentina and Chile, although the majority is found in Mexico and Brazil [8]. Native Americans used the root as a chewing stick, a dye and as an astringent. Many other applications were later devised by the Europeans [9]. Currently, the roots of the *Krameria* species are used in traditional medicine as a remedy for inflammation, diarrhoea, infection and cancer as well as for its photoprotective activity. Because of its many applications, some species are marketed throughout the Americas and Europe [10]. A series of phytochemical studies were performed on the *Krameria* genus by Achenbach *et al.* [11]. They mentioned, and other articles reported, several lignans and some triterpenes as constituents of this genus [11,12,13,14]. *K. pauciflora* is a perennial herb plant. Its roots are used in different regions of Mexico to treat inflammation, cancer and diabetes, and its aerial parts are used to treat gastrointestinal disorders and gastric cancer [15]. Because there are no reports of *in vivo* anti-inflammatory or antidiabetic effects for this species, we evaluated the *in vivo* effect of the roots of *K. pauciflora* on both disorders and its *in vitro* antiradical activity as well as identified its main constituents.

## 2. Results and Discussion

*Krameria pauciflora*, used as an anti-inflammatory and antidiabetic remedy was the object of the present study. *In vivo* evaluations of the effect on carrageenan-induced oedema and glucose levels in rats were carried out and *in vitro* antioxidant activity. Identification of its main constituents was realized.

### 2.1. Anti-inflammatory Assay

**Figure 1:**
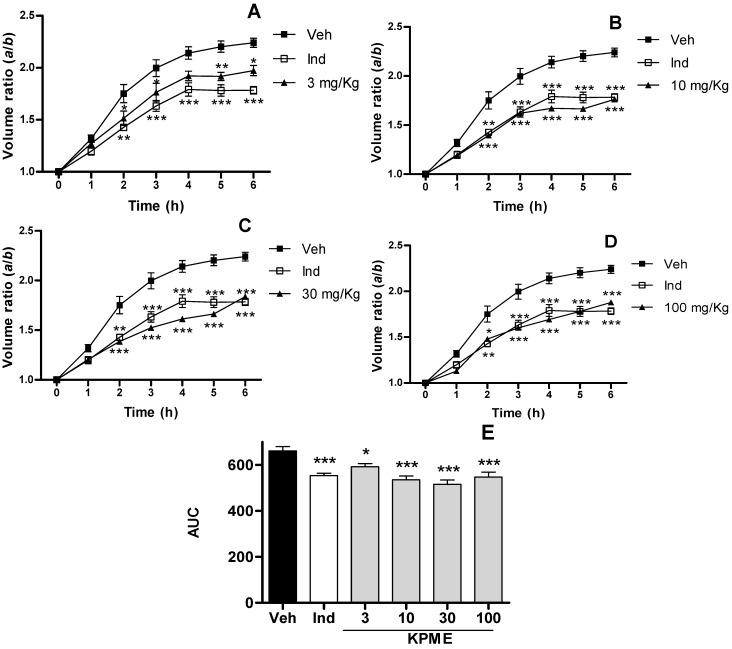
(**a–d**) Course of swelling induced after carrageenan injection presented as volume ratio *a*/*b*, where *a* and *b* are the volumes of right paw after and before carrageenan treatment ± SEM, so each rat is its own control for time = 0, n > 6, treatments are statistically different; (**e**) Anti-inflammatory effect of KPME, data represent the mean of area under curve of volume ratio of hourly determinations 1–6 h ± SEM, n > 6. Veh (vehicle), Ind (indomethacin 10 mg/Kg), 3, 10, 30 and 100 mg/Kg weight. * *p* < 0.05, ** *p* < 0.01, *** *p* < 0.001.

**Figure 2:**
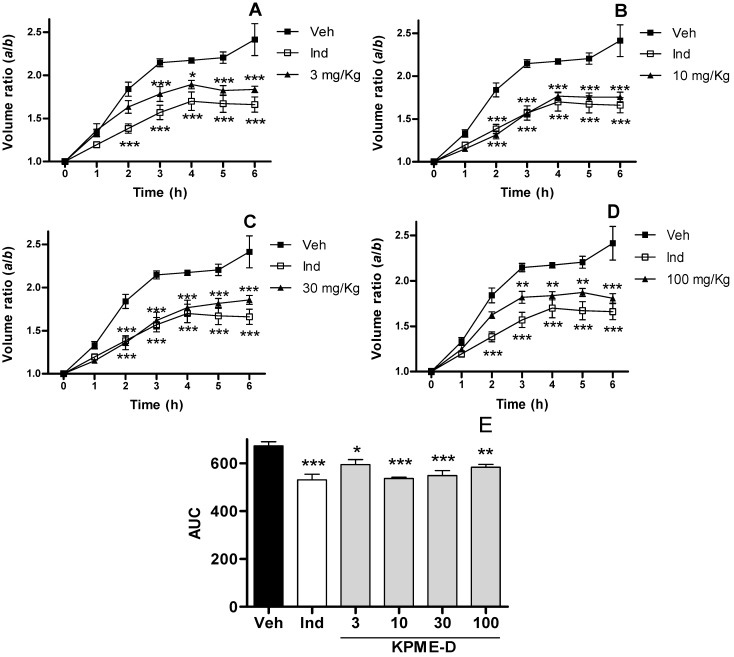
**(a**–**d**) Time-effect curves of KPME-D on carrageenan induced oedema in volume ratio *a*/*b*, where *a* and *b* are the volumes of right paw after and before carrageenan treatment ± SEM, so each rat is its own control for time = 0, n > 6, treatments are statistically different; (**e**) Anti-inflammatory effect of KPME-D on carrageenan induced paw edema. Data represent the mean of area under curve of volume ratio of hourly determinations 1–6 h ± SEM, n > 6. Veh (vehicle), Ind (indomethacin). * *p* < 0.1, ** *p* < 0.01, *** *p* < 0.001.

The carrageenan model of inflammation has been used for a long time because it has presented high predictive activity in human beings. Extracts from *Krameria pauciflora* were evaluated in this model. Comparing the volumes of the right hind paw of each animal showed that the vehicle presented more inflammation than any other treatment. Figure 1 and Figure 2 shows the temporal course of *Krameria pauciflora* methanol extract (KPME) and dichloromethane extract from KPME (KPME-D) respectively with vehicle and indomethacin groups plotted as the volume ratio *versus* time. For clarity, treatments of the extracts are presented as AUC (area under curve, Figure 1e and Figure 2e) of temporal course. KPME at administered doses 3 mg/kg (*p* < 0.05) and 10, 30 and 100 mg/kg (*p* < 0.001) showed anti-inflammatory effects as shown in Figure 1. KPME showed its maximal efficacy at a dose of 30 mg/kg, although all doses, even a dose of 3 mg/kg, presented anti-inflammatory effects similar to indomethacin (10 mg/kg, *p* < 0.001).

KPME-D exhibited an anti-inflammatory effect in all doses tested in this work 3 mg/kg (*p* < 0.05), 10 and 30 mg/kg (*p* < 0.001) and 100 mg/kg (*p* < 0.01), Figure 2. A comparison to indomethacin showed no significant difference to any of the evaluated doses of KMPE-D. Ethyl acetate extract from KPME (KPME-E) showed no activity in this assay (S1).

It is worth highlighting that a 10 mg/kg dose of KPME exhibited an equipotent effect to an equivalent dose of indomethacin. This finding is important because the extract shows the same effect as the pure reference compound (Figure 1b). It is noteworthy that KPME-D, KPME and indomethacin displayed equipotent effects at equivalent doses (10 mg/kg); however, major doses of the extracts did not display significant increase effects. This anti-inflammatory effect could be attributed to the cyclomargenol (**1**) and β-sitosterol (**2**) in the extracts (see Section 2.4). Some sterols and cycloartane-type triterpenes have been reported as anti-inflammatory compounds [16,17] and cancer chemopreventive compounds by Kikuchi *et al*. [18]. Additionally, there are reports that establish this method implies two phases in which several mediators are involved. The first phase (1 h) is mediated by histamine, 5-hydroxytriptamine, serotonin and bradykinin. The production of local prostaglandins derived from COX (cyclooxygenases) activity, especially those of the E series are of particular importance in the second phase (3–5 h). The mechanism of action of the most important non-steroidal anti-inflammatory drugs in actual therapy is inhibition of this phase throughout dismiss COX enzymes activity. KPME and KPME-D presented inhibition of oedema from 2 to 6 h (second phase) which suggest that the extract is probably associated to inhibition of COX enzymes (Figure 1 and Figure 2). Oxidative species has been related to the last phase of inflammation, KPME-E presented antioxidant capacity (Section 2.3) but no anti-inflammatory activity was observed in this work, so this fact supports the suspect that KPME and KPME-D posses anti-inflammatory activity probably due to COX inhibition. KPME-E showed no anti-inflammatory effect because it displayed the same AUC as the vehicle (661 ± 18) for each dose tested in this work (S1).

### 2.2. Antidiabetic Assay

**Figure 3:**
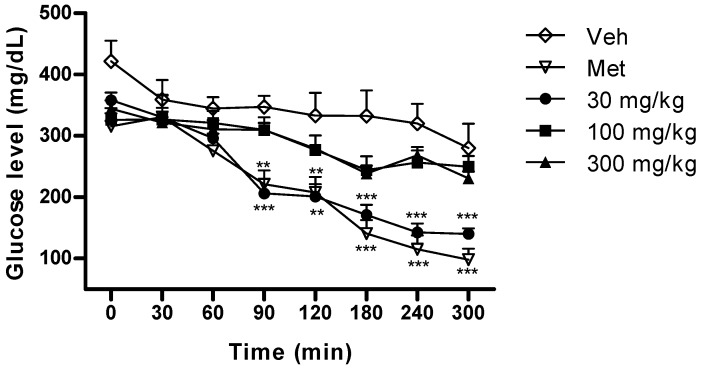
Antihyperglycaemic effect of KPME on STZ induced diabetic rats. Data represents the mean ± SEM, n > 6, Veh (vehicle), Met (metformin, 100 mg/Kg). ** *p* < 0.01, *** *p* < 0.001.

A first approach was attempted in normal rats using glibenclamide as the control compound but no hypoglycaemic effect was observed with any extract (KPME, KPME-D or KPME-E) for the doses evaluated in this work (30, 100 and 300 mg/kg, *p* < 0.05). To explore antihyperglycaemic activity, the assay was then performed with metformin as the reference compound founding no difference comparing vehicle, metformin and extracts in normoglycaemic rats (S2). No effect was observed with KPME-E and KPME-D extracts in STZ (streptozotocin) diabetic rats (data not shown). By the other hand KPME at a 30 mg/kg dose showed an effect (Figure 3, *p* < 0.01) similar to metformin (100 mg/kg, *p* < 0.01).

It should be noted that a small dose of extract (KPME 30 mg/kg) exhibited an equipotent effect to the pure reference compound (Met, 100 mg/kg). The flavonoid content of KPME (see Section 2.4) could explain the antidiabetic use of this species. Catechin-containing beverages administered for medium or long time periods has been reported to improve certain aspects of diabetic conditions, such as obesity, blood pressure, cholesterol levels and insulin secretory ability [19]. A subchronic or chronic study of *K. pauciflora* would be seem promising.

### 2.3. Free Radical Scavenger Determination

*K. pauciflora* demonstrated potential as a radical scavenger (Table 1). Extracts tested in both assays showed the same antioxidant capacity. Antioxidant capacity of the extracts is attributable to the phenolic (flavonoids) content of the species. This finding is valuable because many pathogenic conditions, including diabetes, produce free radicals. Inflammation is mediated by macrophages, leukocytes and neutrophils through oxidative species. Therefore, antioxidants could be useful for anti-inflammation in long term additionally to COX inhibition. Additionally, it is suggested that antioxidant treatments may protect the pancreatic beta cells in animal models with type 2 diabetes [20].

**Table 1:** Radical Scavenger Activity of *K. pauciflora* roots extracts.

	DPPH	ABTS
**KPME**	46.09 ± 2.20	46.09 ± 2.19
**KPME-D**	248.4 ± 1.04	248.4 ± 1.48
**KPME-E**	70.70 ± 1.01	70.71 ± 1.01
**Trolox**	39.14 ± 1.04	38.81 ± 1.03

Data are presented as EC_50_ (μg/mL) ± SEM, n > 5, absorbance of appropriate blank was subtracted of each absorbance value before calculation.

### 2.4. Identification of Compounds

**Figure 4:**
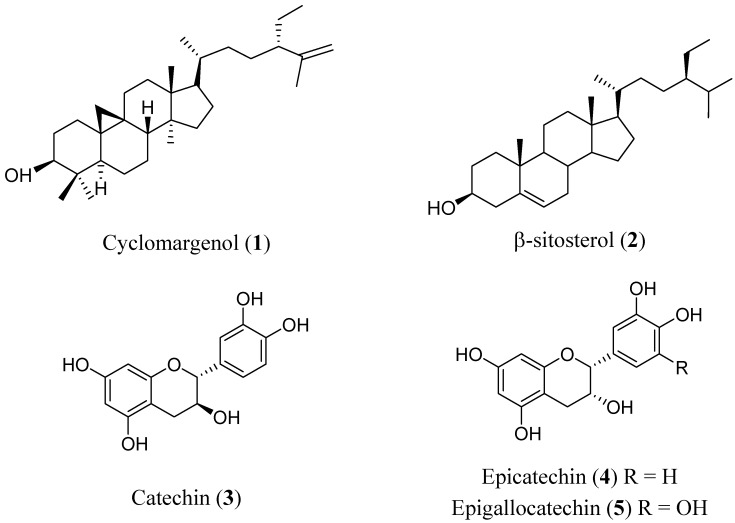
Compounds characterized from the *K. pauciflora* methanol extract.

A phytochemical study was carried out of *K. pauciflora* with conventional methods. KPME was partitioned into KPME-D and KPME-E. A cycloartane-type triterpene (cyclomargenol, **1**), β-sitosterol (**2**) and several fatty acids were identified from 4.5 g of KPME-D extract by mean of spectroscopic and spectrometric data compared with literature (see Section 3.8). Spectroscopic and spectrometric data are available in S3. The compound **1** had been reported as chemopreventive in an *in vivo* model [18] and the sterol **2** had been evaluated as anti-inflammatory in a model of asthma and in other immune-related disorders [21,22]. The flavonoids catechin (**3**), epicatechin (**4**), epigallocatechin (**5**) were identified from 40 g of KPME-E extract (Figure 4). Benefits of catechin type flavonoids ingestion in diabetic people is well known [19]. Constituents of these two extracts are in fact of KPME. This report is the first of these flavonoid-type compounds in this species; catechin or epicatechin gallate were reportedly found in *K. triandra* roots [23].

## 3. Experimental

### 3.1. Plant Material

*Krameria pauciflora* MOC et. Sessé ex DC. roots were collected in Durango, Mexico, in November 2005 by M. C. Ramiro Rios Gómez from Zaragoza Graduate School, National Autonomous University of Mexico (UNAM). A specimen (voucher # 9545) was deposited in the Herbarium at this same Faculty for future reference.

### 3.2. Preparations and Extraction Procedures

The roots of *K. pauciflora* (3.6 kg) were dried at room temperature and powdered. A methanol extract of *K. pauciflora* (KPME) was obtained by macerating vegetal material with methanol at ambient temperature (3 × 4 L × 48 h) and recovered by rotary evaporation (350 g). Then, a portion of the dry methanol extract (200 g) was dissolved in 30% methanol (100 mL) and partitioned into CH_2_Cl_2_ (KPME-D, 3 × 100 mL, 5.8 g) and EtOAc (KPME-E, 3 × 100 mL, 53.1 g). For *in vivo* assays the extracts were dissolved or suspended in an adequate vehicle (Veh): Water for KPME and KPME-E and 1% ethanol in water for KPME-D. Then, these extracts were administered orally (p.o.) in a single logarithmic dose (3, 10, 30 and 100 mg/kg for the inflammation assay and 30, 100 and 300 mg/kg for the diabetes assay) in a volume of 0.2 mL/100 g body weight. *In vitro* assays were carried out in methanol solutions of the extracts at concentrations of 31.25, 62.5, 125, 250, 500 and 1000 μg/mL.

### 3.3. Chemicals and Drugs

Carrageenan, sodium chloride, indomethacin, STZ, glibenclamide, metformin, DPPH (diphenyl-1-picrylhydrazyl), trolox, ABTS (2,2′-azinobis(3-ethylbenzothiazoline-6-sulfonic acid)), ceric ammonium sulphate, vanillin and deuterated solvents for NMR spectra acquisition were purchased from Sigma-Aldrich^®^. Absolute ethanol purchased from Caledon^®^.

### 3.4. Animals

Experiments were performed on male *Wistar* rats weighing 170–250 g from Harlam (Mexico City, Mexico). The rats were housed at a temperature of 22 ± 2 °C in polypropylene cages with metal mesh lids and sawdust bedding. All animals were exposed to a 12 h light/dark cycle. Water and a standard pellet laboratory animal diet were supplied *ad libitum.* The food was removed 12 h prior to experimentation except for STZ-induced diabetic rats. The experiments were performed in accordance with the Mexican Official Norm (NOM-062-ZOO-1999, revised in 2001) and US guidelines (NIH publication #85-23, revised in 1985). Each experiment was carried out between 7:00 and 17:00 h. The rats were acclimated to the laboratory for at least five days before testing. Each rat was used only once and was sacrificed in a CO_2_ chamber immediately after the experiment.

### 3.5. *In Vivo* Anti-Inflammatory Assay (Carrageenan-Induced Paw Oedema)

The anti-inflammatory activity of KPME, KPME-D and KPME-E extracts was determined by carrageenan-induced oedema test in the hind paws of rats as described by Morris [24], with minor modifications. All samples, indomethacin (10 mg/kg,) and vehicle (water and 1% ethanol in water) were administered orally (0.2 mL/100 g weight) at the same time as the carrageenan injection. The rats fasted for 12 h before the experiment and were given free access to water. Then, 50 μL of a 1% carrageenan (10 mg/kg, type IV, lambda) suspension was injected into the plantar side of the right hind paw of each rat. The extracts, KPME, KPME-D and KPME-E, were orally administered at the same time as the injection. The volume of oedema (mL) was measured before and after 1, 2, 3, 4, 5 and 6 h using a plethysmometer (37140 Ugo Basile, Italy). The degree of swelling was evaluated by the ratio *a*/*b*, where *a* and *b* are the volumes of the right hind paw after and before the carrageenan injection, respectively. Indomethacin (Ind, 10 mg/kg) was used as a positive control. The results are presented as the area under the curve (AUC) from the temporal course of time *versus* the volume ratio.

### 3.6. *In Vivo* Antidiabetic Assay

The effect of KPME, KPME-D and KPME-E administration on glucose levels was determined by the glucose-oxidase method using automated equipment in normoglycaemic and STZ-induced diabetic rats. The animals were diabetised with a single i.p. dose of STZ (60 mg/kg). Establishment of a diabetic condition was monitored by measuring glucose plasma levels two days, one week and two weeks after injection. Animals with glucose levels up to 200 mg/kg were considered diabetic. The glucose level of the STZ diabetic rats was measured before and 30, 60, 120, 150, 180, 240 and 300 min after administering the oral extract, metformin (Met, 100 mg/kg) or vehicle. A glucose tolerance curve was determined for the normoglycaemic rats by administering the extracts 25 min after administration of the glucose (2.0 g/kg). All samples, glucose, metformin and vehicle were orally administered, and the glucose levels were measured at the same times as the STZ diabetic groups.

### 3.7. *In Vitro* Antiradical Scavenger Activity

The colorimetric assays were performed in 96-well plates, and the absorbance readings were taken using a Bio-Rad 680^®^ microplate reader. All chemicals were obtained from Sigma-Aldrich^®^ except ethanol as indicated in chemicals and drugs. The DPPH assay was performed according to procedures reported by Huang *et al.* except that the reaction volumes were reduced to fill the wells of a 96-well plate (175 μL of DPPH, 25 μL sample). In brief, reactions of samples (extracts and controls) with DPPH were maintained in dark, the absorbance readings were taken at 490 nm after 30 min [25]. The ABTS assay was carried out as reported by Re *et al.* with minor modifications, such as 20 μL of each sample was mixed with 230 μL of diluted ABTS, and the absorbance of the reaction products was measured after 15 min [26].

### 3.8. Phytochemical Analysis. Purification and Identification of Compounds

Isolation and purification were carried out by conventional phytochemical procedures performed with open column chromatography (CC), medium pressure liquid chromatography using flash silica gel (230–400 mesh) as the stationary phase and radial chromatography using silica gel 60 PF_254_ containing gypsum from Merck^®^. Mobile phases consisted of a mixture of two common solvents (*n*-hexane, dichloromethane, acetone, ethyl acetate and methanol). For medium pressure liquid chromatography, a Büchi^®^ instrument containing a pump controller C-610 and a C-601 pump unit was used. Radial chromatography was carried out using Harrison research^®^ equipment. TLC sheets (Silica gel 60 F_254_, Merck^®^) were used to monitor all chromatographic processes, observed under UV light and subsequently developed using ceric ammonium sulphate or vanillin. KPME-D was purified by CC with *n*-hexane as the initial solvent and then increasing to more polar gradients with ethyl acetate to obtain 5 fractions (A–E). Fraction A was composed of fatty acids and analyzed by GC-MS, hexadecanoic acid, octadecanoic acid, octadecanedioic acid, eicosanoic acid, docosanoic acid, docosanedioic acid, tetracosanoic acid, hexacosanoic acid, 16-hydroxy-hexadecanoic acid, octadecanol and phytol were identified. Fraction B eluted with 95:05 *n*-hex-EtAcO and was further chromatographed using an *n*-hex-acetone 99:01 system with polar gradients. The fractions that eluted with the 97:03 system were then purified by radial chromatography using a 90:10 *n*-hex-acetone system to obtain 24-ethyl-9β,19-cyclo-5α-lanost-25-en-3β-ol (**1**, cyclomargenol 20.8 mg). 24-ethyl-5α-lanost-5-en-3β-ol (**2**, β-sitosterol 40.5 mg) was isolated from fraction C using CC with 80:20 *n*-hex-EtAcO. Fraction D was purified on CC to obtain **1**. KPME-E (40 g) was purified by CC using a mobile phase of *n*-hex-EtAcO 6:4 with polarity gradients of EtAcO, and the collected fractions were grouped into 3 fractions (A-C). Fraction A contained part of fraction B. Fraction B was successively purified by CC using medium pressure liquid chromatography with different mobile phases. The collected fractions contained mixtures of (2*R*,3*S*)-2-(3,4-dihydroxyphenyl)-3,4-dihydro-2*H*-chromene-3,5,7-triol (**3**, catechin), (2*R*,3*R*)-2-(3,4-dihydroxyphenyl)-3,4-dihydro-2*H*-chromene-3,5,7-triol (**4**, epicatechin) and (2*R*,3R)-2-(3,4,5-trihydroxyphenyl)-3,4-dihydro-2*H*-chromene-3,5,7-triol (**5**, epigallocatechin), but no pure compound could be isolated. A mixture of **3** and its epimer, **4**, was collected using radial chromatography with a dichloromethane-methanol 90:10 solvent system. Fraction C contained colorants. The compounds **1**–**5** were identified by analysing their spectroscopic (NMR, IR) and spectrometric (MS) properties and comparing them to the literature (see S3) [27,28,29]. NMR spectra were recorded using a 200 MHz Varian^®^ and 400 MHz Varian-Unity^®^ spectrometer. Samples were dissolved in deuterated solvents purchased from Sigma-Aldrich^®^. IR spectra were obtained in a film consisting of a dichloromethane solution in a NaCl cell with an IR Bruker^®^ Vector 22 device. Mass spectra were recorded on a high resolution JEOL JMS - 700^® ^spectrometer. GC-MS were recorded using a 6890 Agilent^®^ device coupled with a 5973 Agilent^® ^spectrometer.

### 3.9. Statistics

For the *in vivo* assays, the values are reported as the mean of at least 6 animals ± SEM. For the *in vitro* tests, the values are reported as the mean of at least 5 measurements ± SEM. The statistical analyses were performed using one-way ANOVA followed by Dunnett’s *t*-test or Tukey’s multiple comparison or two-way ANOVA with Bonferroni post test using Graphpad Prism version 4.00 for Windows (Graphpad SoftWare, San Diego, CA, USA). A *p* < 0.05 was established as criterion of statistical significance.

## 4. Conclusions

Methanol and dichloromethane extracts of *Krameria pauciflora* roots exhibited *in vivo* anti-inflammatory effects similar to indomethacin when compared at equivalent doses. Furthermore, even at the smallest dose, there are no significant differences between the extracts and the reference compound. The anti-hyperglycaemic *in vivo* effect observed with the methanolic extract of *K. pauciflora* roots at a dose of 30 mg/kg was similar to the effect of a 100 mg/kg dose of metformin. It is important to note that *K. pauciflora* extracts tested in this work presented anti-inflammatory and anti-hyperglycaemic effects similar to the reference compounds at doses smaller than those of the commercial drugs. An *in vitro* radical scavenger capacity was observed for *K. pauciflora* extracts; therefore, this species could be useful for the treatment of inflammatory disorders and to prevent diabetic complications associated with inflammation and/or free radicals. Our study supports the use of this species as an anti-inflammatory and partially supports its use for diabetic patients because of its antioxidant and anti-inflammatory effects.

## Supplementary Materials

Supplementary materials can be accessed at: http://www.mdpi.com/1420-3049/17/1/861/s1.
