# Supplementary material for: Phytochemical Study and Anti-inflammatory, Antidiabetic and Free Radical Scavenger Evaluations of Krameria pauciflora Methanol Extract †
Source: Molecules. 2012 Jan 17;17(1):861–72. doi: 10.3390/molecules17010861 (PMC6268554; doi:10.3390/molecules17010861)
Supplement: Supplementary file 1 [file molecules-17-00861-s001.doc]

Phytochemical Study and Anti-inflammatory, Antidiabetic and Free Radical Scavenger Evaluations of *Krameria pauciflora* Methanol Extract

**M. Ángeles Ramírez-Cisneros, Maria Yolanda Rios, Myrna Déciga-Campos and
A. Berenice Aguilar-Guadarrama ***

**S1.** Anti-inflammatory effect of KPME-E on carrageenan induced paw edema. Data represent the mean of area under curve of volume ratio of hourly determinations 1–6 h ± SEM, n > 6. Veh (vehicle), Ind (indomethacin 10 mg/Kg), 3, 10, 30 and 100 mg/Kg weigh, * *p* < 0.05.

**S2.** Effect of KPME on glucose tolerance test in normoglycaemic animals. Data represents the mean of 6 animals ± SEM. Veh (vehicle), Met (Metformin). No statistical difference was observed for any treatment respect to vehicle group (*p* < 0.05).

**S3**. Spectroscopic and spectrometric data of compounds **1**–**5**.

*Cyclomargenol* (**1**): ^1^H-NMR (400 MHz, CDCl_3_) δ: 0.33 (CH, d, 4.0 Hz H-19), 0.55 (CH, d, 4.0 Hz, H-19), 0.85 (CH_3_, s, H-21), 0.80 (CH_3_, s, H-32), 0.81 (CH_3_, s, H-28 or H-29), 0.95 (CH_3_, s, H-18), 0.96 (CH_3_, s, H-30), 1.57 (CH_3_, m, H-26), 3.28 (CH, m, H-3), 4.65 (CH, d, 2.4 Hz, H-27), 4.73 (CH, d, 2.4 Hz, H-27). ^13^C-NMR (100 MHz, CDCl_3_), δ: 12.4 (CH_3_, C-32), 79.0 (CH, C-3), 111.6 (CH_2_, C-27), 147.7 (C, C-25). HREI obs. 454.4143 (calc. 454.4175).

β-*Sitosterol* (**2**): ^1^H-NMR (200 MHz, CDCl_3_) δ: 0.80 (CH_3_, t, 7.8, 15.78 Hz, H-29), 0.92 (CH_3_, d, 7.2 Hz, H-27), 0.94 (CH_3_, d, 6.7 Hz, H-26), 0.98 (CH_3_, d, 6.1 Hz, H-21), 0.99 (CH_3_, s, H-18), 1.09 (CH_3_, s,
H-19), 3.52 (CH, m, H-3), 5.13 (CH, s, H-6). ^13^C-NMR (50 MHz, CDCl_3_) δ: 12.1 (CH_3_, C-18), 12.2 (CH_3_, C-29), 19.1 (CH_3_, C-21), 19.3 (CH_3_, C-26), 19.7 (CH_3_, C-19), 20.1 (CH_3_, C-27), 21.4 (CH_2_,
C-11), 23.3 (CH, C-28), 24.6 (CH_2_, C-15), 26.3 (CH_2_, C-23), 28.5 (CH_2_, C-16), 29.4 (CH, C-25), 31.9 (CH_2_, C-2), 32.2 (CH_2_, C-7), 32.2 (CH_2_, C-8), 34.2 (CH_2_, C-22), 36.4 (C, C-10), 36.8 (CH, C-20), 37.5 (CH_2_, C-1), 42.6 (C, C-13), 46.08 (CH, C-24), 50.4 (CH, C-9), 56.3 (CH, C-14), 57.0 (CH, C-14), 72.0 (CH, C-3), 121.9 (CH, C-6), 140.9 (C, C-5). HREI obs. 415.3948 (calc. 415.3940)

*Catechin* (**3**): ^1^H-NMR (400 MHz, CDCl_3_) δ: 2.50 (CH, dd, 2.0, 8.0 Hz, H-4), 2.84, (CH, dd, 5.6, 16.0 Hz, H-4) 3.97 (CH, ddd, 8.0, 8.0, 2.0 Hz, H-3), 4.57 (CH, d, 8.0 Hz, H-2), 5.87 (CH, d, 2.4 Hz, H-8), 6.72 (CH, dd, 2.0, 8.0 Hz, H-6′), 6.76 (CH, d, 8.0 Hz, H-5′), 6.84 (CH, d, 2.0 Hz, H-2′). ^13^C-NMR (100 MHz, CDCl_3_) δ: 28.6 (CH_2_, C-4), 69.0 (CH, C-3), 83.0 (CH, C-2), 95.8 (CH, C-8), 96.6 (CH, C-6), 101.1 (C, C-10), 115.5 (CH, C-2′), 116.3, (CH, C-5′), 120.2 (CH, C-6′), 132.5 (C, C-1′), 146.0 (C, C-3′), 146.0 (C, C-4′), 157.1 (C, C-9), 157.7 (C, C-5), 158.0 (C, C-7).

*Epicatechin* (**4**): ^1^H-NMR (400 MHz, CDCl_3_) δ: 2.50 (CH, m, H-4), 2.74, (CH_2_, dd, 2.8, 12.8 Hz, H-4), 4.17 (CH, m, H-3), 4.84 (CH, m, H-2*), 5.81 (CH, d, 2.4 Hz, H-8*), 6.67 (CH, dd, 2.0, 8.0 Hz, H-6′), 6.76 (CH, d, 8.0 Hz, H-5′), 6.97 (CH, d, 2.0 Hz, H-2′). ^13^C-NMR (100 MHz, CDCl_3_) δ: 29.3 (CH_2_,
C-4), 67.7 (CH, C-3), 80.1 (CH, C-2), 92.2 (CH, C.8), 96.8 (CH, C-6), 100.4 (C, C-10), 115.6 (CH,
C-2′), 116.2, (CH, C-5′), 119.6 (CH, C-6′), 132.5 (C, C-1′), 146.0 (C, C-3′), 146.0 (C, C-4′), 157.0 (C, C-9), 157.6 (C, C-5), 157.9 (C, C-7).

*Epigallocatechin* (**5**): ^1^H-NMR (400 MHz, CDCl_3_) δ: 2.50 (CH, m, H-4), 2.74 (CH_2_, dd, 2.8, 12.8 Hz, H-4), 4.18 (CH, m, H-3), 4.84 (CH, m, H-2*), 5.81 (CH, d, 2.4 Hz, H-8*), 6.53 (CH, d, 3.6 Hz, H-6′ y H-2′). ^13^C-NMR (100 MHz, CDCl_3_) δ: 29.3 (CH_2_, C-4), 67.7 (CH, C-3), 80.1 (CH, C-2), 96.2 (CH, C.8), 96.8 (CH, C-6), 100.4 (C, C-10), 107.4 (CH, C-6′), 107.5 (CH, C-2′), 132.5 (C, C-1′), 132.5 (C, C-4′), 146.8 (CH, C-5′), 147.3 (C, C-3′), 149.6 (C, C-9), 151.5 (C, C-5), 151.5 (C, C-7).

* Note that catechins had been identified in mix according their ^13^C-NMR data. Epicatechin and epigallocatechin ^1^H assignment is difficult due to overlapping of signals.
